# Supplementary material for: Adherence to COVID-19 preventive practice and associated factors among pregnant women in Gondar city, northwest Ethiopia, 2021: Community-based cross-sectional study
Source: PLoS One. 2022 Mar 2;17(3):e0264237. doi: 10.1371/journal.pone.0264237 (PMC8890631; doi:10.1371/journal.pone.0264237)
Supplement: S1 Questionnaire — (DOCX) [file pone.0264237.s001.docx]

**English version Questionnaire**

Part : Socio demographic characteristics of a pregnant women

| S.N | Question | Answers | skip |
| --- | --- | --- | --- |
| 101 | What is your age? | __________years |  |
| 102 | What is your Religion? | 1. Orthodox 2. Muslim 3. Protestant 4. Others(specify) _____ |  |
| 103 | What is your level of education? | 1. Unable to read and write 2. Able to read and write 3. Elementary(1-8) 4. Secondary school(9-12) 5. College and above |  |
| 104 | What is your Occupation? | 1. House wife 2. Farmer 3. Civil servant 4. Private Business 5. Others(specify) ______ |  |
| 105 | What is your current marital status? | 1. Married 2. Single 3. Divorced 4. Widowed | If the answer is 2,3&4 skip to Q 108 |
| 106 | What is your Husband’s level of education? | 1. Unable to read and write 2. Able to read and write 3. Elementary(1-8) 4. Secondary school(9-12) 5. College and above |  |
| 107 | What is your Husband Occupational status? | 1. Farmer 2. Civil Servant 3. Private business 4. Others(specify) ______ |  |
| 108 | What is the number of family living together? | __________ |  |
| 109 | What is the average family monthly income? | _________ ETH birr |  |

**Part II: Obstetrics and reproductive related questions**

| 201 | What is your Gravidity? | _________ |  |
| --- | --- | --- | --- |
| 202 | What is your number of Alive children? | _________ |  |
| 203 | Did you had History of Previous adverse pregnancy outcome? | 1. Yes 2. No |  |
| 204 | Do you have ANC follow-up? | 1. Yes 2. No | If no skip to Q 206 |
| 205 | If yes, Number of ANC follow-up | _________ |  |
| 206 | What is your condition of pregnancy? | 1. Planned 2. Unplanned |  |
| 207 | Do you have History of chronic illness? | 1. Yes 2. No |  |

**Part III: Knowledge about COVID-19**

| 301 | Have you Ever heard about covid-19 | 1. Yes 2. No |  |
| --- | --- | --- | --- |
| 302 | Covid-19 is Viral disease | 1. Yes 2. No 3. Neutral |  |
| 303 | Do you think Respiratory droplets and close contact are the main transmission route? | 1. Yes 2. No 3. Neutral |  |
| 304 | Do you think all people are generally susceptible to COVID-19 | 1. Yes 2. No 3. Neutral |  |
| 305 | Dry cough is a symptom of Covid-19? | 1. Yes 2. No 3. Neutral |  |
| 306 | Fever is a symptom of Covid-19? | 1. Yes 2. No 3. Neutral |  |
| 307 | Headache is a symptom of Covid-19? | 1. Yes 2. No 3. Neutral |  |
| 308 | Sore thorat is a symptom of Covid-19? | 1. Yes 2. No 3. Neutral |  |
| 309 | Runny nose is a symptom of Covid-19? | 1. Yes 2. No 3. Neutral |  |
| 310 | Difficulty of breathing is a symptom of Covid-19? | 1. Yes 2. No 3. Neutral |  |
| 311 | Do you think Stay at home and wearing face mask can prevent COVID-19 | 1. Yes 2. No 3. Neutral |  |
| 312 | Do you think People with co-existing disease has poor prognostic outcome | 1. Yes 2. No 3. Neutral |  |
| 313 | Can Person with COVID-19 transmit the virus to others without developing sign | 1. Yes 2. No 3. Neutral |  |
| 314 | Do you think that Pregnant women are high risk to COVID-19 than others | 1. Yes 2. No 3. Neutral |  |

**Part IV: Practice questions**

| 401 | Did you Wash hands frequently with water and soap or with sanitizer in the past 2 weeks? | 1. Yes 2. No |  |
| --- | --- | --- | --- |
| 402 | Do you Avoid touching eyes, nose and mouth with unwashed hand in the past 2 weeks? | 1. Yes 2. No |  |
| 403 | Are you Cover the mouth and nose during coughing and sneezing in the past 2 weeks? | 1. Yes 2. No |  |
| 404 | Are you regularly Wear face mask in public in the past 2 weeks? | 1. Yes 2. No |  |
| 405 | Have you Stay at home or in door to protect the spread of COVID-19 in the past 2 weeks? | 1. Yes 2. No |  |
| 406 | Did you Maintain 1 meter distance from others in the past 2 weeks? | 1. Yes 2. No |  |

**Thank you!!!**
