# Supplementary material for: Adherence to COVID-19 preventive practice and associated factors among pregnant women in Gondar city, northwest Ethiopia, 2021: Community-based cross-sectional study
Source: PLoS One. 2022 Mar 2;17(3):e0264237. doi: 10.1371/journal.pone.0264237 (PMC8890631; doi:10.1371/journal.pone.0264237)
Supplement: Attachment — Submitted filename: PONE-D-21-40668_reviewer.pdf.pdf [file pone.0264237.s003.pdf]

PLOS ONE

Adherence with COVID-19 preventive practice and associated factors among pregnant women in Gondar city, northwest Ethiopia, 2021: Community based cross -sectional study

--Manuscript Draft--

**Manuscript Number:**	PONE-D-21-40668
**Article Type:**	Research Article
**Full Title:**	Adherence with COVID-19 preventive practice and associated factors among pregnant women in Gondar city, northwest Ethiopia, 2021: Community based cross -sectional study
**Short Title:**	Adherence with COVID-19 preventive practice and associated factors among pregnant women
**Corresponding Author:**	Wubedle Zelalem Temesgan, Msc<br>University of Gondar<br>Gondar, Amhara region ETHIOPIA
**Keywords:**	Pregnant women, adherence, preventive practice, COVID-19, Ethiopia
**Abstract:**	Background: Coronavirus disease 2019 (COVID-19) causes more than five million deaths worldwide. Pregnant women are at high risk for infection due to the physiologic change in the immune and cardiopulmonary system and also it increases the risk of severe disease, intensive care unit admission, and receive mechanical ventilation when compared with non-pregnant women. It is associated with adverse maternal and neonatal outcomes. So pregnant women need to have adhered to preventive measures to prevent COVID-19 related consequences. Therefore this study aimed to assess adherence with COVID-19 preventive practice and associated factors among pregnant women in Gondar city, northwest Ethiopia.<br>Methods: A community-based cross-sectional study was conducted from July 1 st to 30 th , 2021, in Gondar city. A cluster sampling technique was employed to select 678 pregnant women. Data were collected using a pre-tested, face-to-face interviewer-administered questionnaire. Data were entered into EPI DATA version 4.6 and exported to SPSS version 25 for analysis. Both bivariable and multivariable logistic regression analysis was fitted to identify associated factors. Adjusted odds ratio with a 95% confidence interval was used to report the association between covariates and the outcome variable.<br>Results: T he prevalence of good adherence with COVID-19 preventive practice was 44.8% (95% CI: 41.3, 48.7). maternal age (≤24 years) (AOR = 2.89, 95% CI: 1.37, 6.10), maternal education (secondary school) (AOR = 2.95, 95% CI: 1.58, 5.53) and (college and above) (AOR = 4.57,95% CI: 2.42, 8.62), having ANC follow up (AOR = 2.95, 95% CI: 1.35, 6.46) and adequate knowledge towards COVID-19 (AOR = 1.70, 95% CI: 1.20, 2.41) were significantly associated with good adherence to COVID-19 preventive practice.<br>Conclusion: In this study, adherence towards COVID-19 preventive practice in pregnant women is low. Hence, it is important to strengthen women's awareness about COVID-19 through different media and health education. In addition, empowering women to attain ANC and special consideration should be given to women who had no formal education.
**Order of Authors:**	Wubedle Zelalem Temesgan, Msc
	Mastewal Belayneh Aklil, Msc
	Henok Solomon Yacob, BSc
	Esubalew Tsega Mekonnen, BSc
	Elias Derso Tegegne, BSc
	Esubalew Binega Tesfa, BSc
	Eshetie Melkie Melese, BSc
	Tewodros zelalem Seyoum, assistant professor

Powered by Editorial Manager® and ProduXion Manager® from Aries Systems Corporation

**Additional Information:**	
**Question**	**Response**
**Financial Disclosure**<br><br>Enter a financial disclosure statement that describes the sources of funding for the work included in this submission. Review the submission guidelines for detailed requirements. View published research articles from *PLOS ONE* for specific examples.<br><br>This statement is required for submission and **will appear in the published article** if the submission is accepted. Please make sure it is accurate.<br><br>**Unfunded studies**<br>Enter: *The author(s) received no specific funding for this work.*<br><br>**Funded studies**<br>Enter a statement with the following details:<br>• Initials of the authors who received each award<br>• Grant numbers awarded to each author<br>• The full name of each funder<br>• URL of each funder website<br>• Did the sponsors or funders play any role in the study design, data collection and analysis, decision to publish, or preparation of the manuscript?<br>• **NO** - Include this sentence at the end of your statement: *The funders had no role in study design, data collection and analysis, decision to publish, or preparation of the manuscript.*<br>• **YES** - Specify the role(s) played.<br><br>* typeset	No fund was received for this work
**Competing Interests**<br><br>Use the instructions below to enter a competing interest statement for this submission. On behalf of all authors, disclose any competing interests that could be perceived to bias this work—acknowledging all financial support and any other relevant financial or non-financial competing interests.	The authors declared no competing interest

This statement is required for submission and **will appear in the published article** if the submission is accepted. Please make sure it is accurate and that any funding sources listed in your Funding Information later in the submission form are also declared in your Financial Disclosure statement.<br><br>View published research articles from *PLOS ONE* for specific examples.<br><br>**NO authors have competing interests**<br><br>Enter: *The authors have declared that no competing interests exist.*<br><br>**Authors with competing interests**<br><br>Enter competing interest details beginning with this statement:<br><br>*I have read the journal's policy and the authors of this manuscript have the following competing interests: [insert competing interests here]*<br><br>* typeset	
**Ethics Statement**<br><br>Enter an ethics statement for this submission. This statement is required if the study involved:<br><br>• Human participants<br>• Human specimens or tissue<br>• Vertebrate animals or cephalopods<br>• Vertebrate embryos or tissues<br>• Field research<br><br>Write "N/A" if the submission does not require an ethics statement.<br><br>General guidance is provided below. Consult the submission guidelines for detailed instructions. **Make sure that all information entered here is included in the**	university of Gondar ethical review board.

**Methods section of the manuscript.**<br><br>**Format for specific study types**<br><br>**Human Subject Research (involving human participants and/or tissue)**<br>• Give the name of the institutional review board or ethics committee that approved the study<br>• Include the approval number and/or a statement indicating approval of this research<br>• Indicate the form of consent obtained (written/oral) or the reason that consent was not obtained (e.g. the data were analyzed anonymously)<br><br>**Animal Research (involving vertebrate animals, embryos or tissues)**<br>• Provide the name of the Institutional Animal Care and Use Committee (IACUC) or other relevant ethics board that reviewed the study protocol, and indicate whether they approved this research or granted a formal waiver of ethical approval<br>• Include an approval number if one was obtained<br>• If the study involved *non-human primates*, add *additional details* about animal welfare and steps taken to ameliorate suffering<br>• If anesthesia, euthanasia, or any kind of animal sacrifice is part of the study, include briefly which substances and/or methods were applied<br><br>**Field Research**<br><br>Include the following details if this study involves the collection of plant, animal, or other materials from a natural setting:<br>• Field permit number<br>• Name of the institution or relevant body that granted permission	
**Data Availability**<br><br>Authors are required to make all data underlying the findings described fully available, without restriction, and from the time of publication. PLOS allows rare exceptions to address legal and ethical concerns. See the PLOS Data Policy and FAQ for detailed information.	Yes - all data are fully available without restriction

Powered by Editorial Manager® and ProduXion Manager® from Aries Systems Corporation

A Data Availability Statement describing where the data can be found is required at submission. Your answers to this question constitute the Data Availability Statement and **will be published in the article**, if accepted.<br><br>**Important:** Stating 'data available on request from the author' is not sufficient. If your data are only available upon request, select 'No' for the first question and explain your exceptional situation in the text box.<br><br>Do the authors confirm that all data underlying the findings described in their manuscript are fully available without restriction?	
**Describe where the data may be found in full sentences. If you are copying our sample text, replace any instances of XXX with the appropriate details.**<br><br>• If the data are **held or will be held in a public repository**, include URLs, accession numbers or DOIs. If this information will only be available after acceptance, indicate this by ticking the box below. For example: *All XXX files are available from the XXX database (accession number(s) XXX, XXX.*).<br>• If the data are all contained **within the manuscript and/or Supporting Information files**, enter the following: *All relevant data are within the manuscript and its Supporting Information files.*<br>• If neither of these applies but you are able to provide **details of access elsewhere**, with or without limitations, please do so. For example:<br><br>*Data cannot be shared publicly because of [XXX]. Data are available from the XXX Institutional Data Access / Ethics Committee (contact via XXX) for researchers who meet the criteria for access to confidential data.*<br><br>*The data underlying the results*	All relevant data are within the manuscript and its Supporting information files.

presented in the study are available from (include the name of the third party and contact information or URL).<br>• This text is appropriate if the data are owned by a third party and authors do not have permission to share the data.<br><br>* typeset	
Additional data availability information:	

Manuscript

Click here to access/download;Manuscript;Manuscript final.edited.docx

Original research article

Adherence ~~with~~ to COVID-19 preventive practice and associated factors among pregnant women in Gondar city, northwest Ethiopia, 2021: Community-based cross-sectional study

Authors

Wubedle Zelalem Temesgan (WZT)[1*], Mastewal Belayneh Aklil (MBA)[1], Henok Solomon Yacob (HSY)[2], Esubalew Tsega Mekonnen (ETM)[2], Elias Derso Tegegne (EDT)[2], Esubalew Binega Tesfa (EBT)[2], Eshetie Melkie Melese (EMM)[2], Tewodros Seyoum (TS)[1]

Affiliation:

[1]Department of Clinical Midwifery, School of Midwifery, College of Medicine and Health Sciences, University of Gondar, Gondar, Ethiopia

[2]Department of general midwifery, School of Midwifery, College of Medicine and Health Sciences, University of Gondar, Gondar, Ethiopia

Corresponding author: Wubedle Zelalem Temesgan

Emails of authors:

WZT: wubedlezelalem@gmail.com

MBA: mastewalbelayneh@gmail.com

HSY: henoksolomon52@gmail.com

ETM: tsegabesu2124@gmail.com

EDT: dersoelias@gmail.com

EBT: esubalewbinega21@gmail.com

EMM: eshetiemelkie07@gmail.com

TS: tedysha@gmail.com

Abstract

Background: Coronavirus disease 2019 (COVID-19) causes more than five million deaths worldwide. Pregnant women are at high risk for infection due to the physiologic change in the immune and cardiopulmonary system and also it increases the risk of severe disease, intensive care unit admission, and receive mechanical ventilation when compared with non-pregnant women. It is associated with adverse maternal and neonatal outcomes. So pregnant women need to have adhered to preventive measures to prevent COVID-19 related consequences. Therefore this study aimed to assess adherence with COVID-19 preventive practice and associated factors among pregnant women in Gondar city, northwest Ethiopia.

Methods: A community-based cross-sectional study was conducted from July 1st to 30th, 2021, in Gondar city. A cluster sampling technique was employed to select 678 pregnant women. Data were collected using a pre-tested, face-to-face interviewer-administered questionnaire. Data were entered into EPI DATA version 4.6 and exported to SPSS version 25 for analysis. ~~Both bivariable~~ simple and ~~multivariable~~ multiple logistic regression analysis was fitted to identify associated factors. Adjusted odds ratio with a 95% confidence interval was used to report the association between covariates and the outcome variable.

It is a multivariate model if the response é multivariate! When you have only 1 explanatory variable, it is simple; with several explanatory variables, it is multiple regression model.

Results: The prevalence of good adherence with COVID-19 preventive practice was 44.8% (95% CI: 41.3, 48.7). maternal age (≤24 years) (AOR = 2.89, 95% CI: 1.37, 6.10), maternal education (secondary school) (AOR = 2.95, 95% CI: 1.58, 5.53) and (college and above) (AOR = 4.57,95% CI: 2.42, 8.62), having ANC follow up (AOR = 2.95, 95% CI: 1.35, 6.46) and adequate knowledge towards COVID-19 (AOR = 1.70, 95% CI: 1.20, 2.41) were significantly associated with good adherence to COVID-19 preventive practice.

Conclusion: In this study, adherence towards COVID-19 preventive practice in pregnant women is low. Hence, it is important to strengthen women's awareness about COVID-19 through different media and health education. In addition, empowering women to attain ANC and special consideration should be given to women who had no formal education.

Keywords: Pregnant women, adherence, preventive practice, COVID-19, Ethiopia

Introduction

Coronavirus disease 2019 (COVID-19) is a novel virus that is caused by severe acute respiratory syndrome coronavirus 2 (SARS-CoV-2) first detected at the end of December 2019, in Wuhan city china (1,2). As COVID-19 rapidly distribute throughout the world, World Health Organization (WHO) declared it as a public health emergency of international concern on January 30, 2020 (3). Following this, due to the continual rise of COVID-19 related cases and deaths, WHO declared the disease a global pandemic on, March 11, 2020 (4–6). The Ethiopian government announced the first case was detected on March 13, 2020, and currently, more than six thousand deaths are recorded (7). Please, include the population of Ethiopia for the public to know.

Globally, COVID-19 had spread to over 219 countries and has influenced different devastating problems to the human race including mortality and morbidity, and social and economic crisis (7,8). As of 20 November 2021 report, confirmed COVID-19 cases were 257,123,909, deaths 5,158,990 and 232,181,236 cases were recovered from COVID-19 in the world, and 8,654,048 cases, 221,941 deaths and 8,035,451 cases were recovered from COVID-19 in Africa and 369,867 cases , 6,662 deaths and 346,547 recovery were recorded in Ethiopia (9).

World Health Organization (WHO) has recommended several preventive measures to curve down the spread of the disease like regular handwashing with water and soap, social distancing, covering masks? the mouth while coughing and sneezing, avoiding touching eyes, nose, and mouth (10). The government of Ethiopia has also engaged in different activities to halt the spread of the virus in the country like Community mobilization, public awareness creation, isolation, compulsory quarantine, and treatment, strict passenger screening, house to house screening, and scaling up diagnostic tests and treatment centers (11).

Every person is at risk of becoming infected with COVID-19. However, Pregnant women are at high risk for infection ~~due to~~ the physiologic change in the immune and cardiopulmonary system during pregnancy makes them more susceptible to SARS-CoV-2 infection when compared to the general population (12,13). Centre for Disease Control and Prevention (CDC) report showed that pregnant women with COVID-19 infection are at increased risk of severe disease, intensive care unit admission, and receive mechanical ventilation when compared ~~with~~ non-pregnant patients (12,14,15). Moreover, it is associated with maternal and neonatal complications like miscarriage,

preterm births, intrauterine growth restriction, fetal distress, and preeclampsia (12,16,17). A study showed that COVID -19 can pass directly from mother to fetus during pregnancy (18). There is also evidence that revealed that during the pandemic the risk of perinatal anxiety, depression, and domestic violence also increase in pregnant women (19). Even though pregnant women have not been included in any COVID-19 vaccine clinical trials, the American College of Obstetricians and Gynecologists and the Society for Maternal-Fetal Medicine recommend that pregnant women can take COVID-19 vaccines if they prefer to be vaccinated (20–22).

Although the spread of COVID -19 increases every day in Ethiopia, the vaccine is not accessible for pregnant women. so pregnant women need to have adhered to preventive measures set by the Ministry of Health (MoH) to decrease the likelihood of being infected by the virus. This reduces maternal and neonatal morbidity and mortality. In Ethiopia, researches addressing the highly vulnerable pregnant women are scarce, particularly in the study area. Therefore, this study aimed to assess adherence with COVID-19 preventive practice and associated factor among pregnant women in Gondar city, northwest Ethiopia.

Methods and materials

Study design and setting

A community-based cross-sectional study was conducted in Gondar city from July 1st to 30th, 2021. Gondar city is located 166 km far from Bahirdar, the capital city of Amhara regional state, and 750 km northwest of Addis Ababa, the capital city of Ethiopia. According to the Population projection of Ethiopia for all regions at the woreda level from 2014 - 2017, the total population of the town was estimated to be 306,246. Among these, 156,276 of the population are females (23). Currently, it has 1 governmental comprehensive specialized hospital, 8 governmental health centers, 22 health posts, 1 private primary hospital, and 1 general hospital serving the population of the city and catchment area.

Study population and eligibility criteria

The study population were all pregnant women in the selected clusters of Gondar city during the data collection period and residing in the study area for at least six months before the data collection period.

Sample size determination and sampling procedure

The sample size was determined by using a single population proportion formula by considering a proportion of good practice 47.6 (24), level of confidence 95%, margin of error 5%, Thus, the sample size (n) $= \frac{(Z\alpha/2)^2 p(1-p)}{d^2} \frac{= (1.96)^2 0.476(1-0.476)}{(0.05)2}$ = 384. After considering a design effect of 1.5, and a non-response rate of 10 %. The total sample size was 634. Gondar city has 22 kebeles and five kebeles were selected by a lottery method. All eligible participants in the selected cluster were included in the study, making the final sample size of 678.

Variables of the study

Adherence with COVID-19 preventive practice was the outcome variable whereas, maternal age, religion, maternal educational status, maternal occupation, marital status, husband educational status, husband occupation, number of families living together, average monthly income, gravidity, number of alive children, having ANC visit, number of ANC visit, condition of pregnancy, history of previous adverse pregnancy outcome, history of chronic illness and knowledge about COVID- 19 preventive practice were independent variable of the study.

Operational definitions and measurements

Adherence to COVID -19 preventive practice: It was measured by using 6 item questions derived from WHO recommendation on prevention measures against COVID -19 and participants were asked to answer their practice in the last 14 days. Each correct response weighed 1 point and 0 for an incorrect response. Participants who scored 100% on the practice of COVID-19 preventive measures were considered as having good adherence with COVID- 19 preventive practice. Whereas, participants who scored < 100% were considered as having poor adherence with COVID- 19 preventive practice (25,26).

Knowledge: - It was measured by using 14 item questions that include clinical presentation, risk factors, prognosis, and prevention measures of COVID-19. Each correct response weighed 1 point and 0 for an incorrect response. Participants who scored ≥65% of knowledge questions were considered as having adequate knowledge and those who scored < 65% were considered as having inadequate knowledge (26).

Data collection tools & procedures

The data collection tool was developed by reviewing related literature (24,26–28). Data were collected using semi-structured, pretested questionnaires through a face-to-face interview. The questionnaire contains socio-demographic characteristics, obstetric-related variables, and knowledge and preventive practice of COVID-19 related questions. Five BSc midwives data collectors and one MSc clinical midwife supervisor have participated in the data collection process .were recruited for data collection and supervision respectively.

Data quality control measures

The questionnaire was prepared in English and then translated into the local language Amharic with the assistance of language experts then translated back to English to maintain consistency of the tool. Before the actual data collection, the questionnaire was pretested on 5% of the total sample size at koladiba district to check the response, language clarity, and appropriateness of the questionnaire. One-day training was given for data collectors and supervisor about the aim of the study, contents of the tool, and techniques of data collection. In addition, the data collectors and supervisor were informed regarding important precautions to be taken to prevent COVID-19 infection. During data collection, data collectors were supervised for any difficulties. The consistency and completeness of the data were checked by the data collectors and supervisor and the incomplete data were discarded before data entry.

Data processing & analysis

The data were checked, coded, and entered into Epi data version 4.6 and then exported to SPSS version 25 for analysis. Descriptive statistics like frequencies, percentages, mean and standard deviation were computed. ~~Bivariable~~ Simple logistic regression was done to identify the association between independent and dependent variables, variables ~~having a p-value~~ with p-values less less than 0.2 were entered into ~~multivariable~~ the multiple logistic regression ~~analysis~~ model. Adjusted odds ratio with 95% CI and a ~~p-value of ≤ 0.05 was used to determine~~ the level of significance. was 5% for all tests.

no p-value was used to determine any level...

Ethical consideration

Ethical clearance was obtained from the University of Gondar ethical review committee. A supportive letter was obtained from selected kebeles administrates. Written informed consent was taken from each study participant after a clear explanation of the aim of the study. Study

participants were also informed that they had the full right to withdraw from the interview at any time.

Results

Socio-demographic characteristics of study participants

In this study, a total of 663 women were included in the analysis making a response rate of 97.8%. The age of the participants ranges from19 to 44 years with a mean age of 31.25 (±5.525 SD) years. More than half (57.5%) of the respondents were in the age group of 25-34 years. Nearly two-thirds (66.4%) of study participants were Orthodox Christians in religion. The majority (94%) of the study participants were married. Regarding husband's educational status nearly one-third (31.5%) of their husband had attended secondary school **(Table 1)**.

Obstetric and reproductive characteristics of study participants

Among the total study participants, more than three-fourths (79.2%) and the majority (93.4%) of them were multigravida in their gravidity and had ANC follow up respectively. Regarding the condition of pregnancy 601 (90.6%) of the pregnancy was planned **(Table 2).**

What is ANC?

Knowledge about COVID-19

From the total study participants, 422 (63.7%) had adequate knowledge about COVID-19. Almost all respondents heard about COVID-19. More than half (52.3%) and (53.5%) of pregnant women said that sore throat and runny nose are a symptom of COVID-19 respectively. Five hundred twenty-seven (79.48 %) respondents know that pregnant women are at high risk for COVID-19 than other populations **(Table 3).**

have higher risk

Adherence with COVID-19 preventive practice

The finding of this study showed that only 44.8% (95% CI: 41.3, 48.7) of the participants had good adherence to COVID-19 preventive practice (**Figure 1**). About three-fourths (74.1%) and majority (90.3%) of participants wash hands with water and soap or with alcohol-based sanitizer and wear a face mask in public to decrease the spread of infection respectively **(Table 4).**

Factors affecting adherence with COVID -19 preventive practice

analysis with only one explanatory variable

or simple logistic regression:

In the ~~bi-variable analysis~~: maternal age, maternal educational status, marital status, number of families living together, average monthly income, gravidity, number of alive children, ANC follow

up and knowledge about COVID-19 were significantly associated with COVID-19 preventive practice adherence at a p-value <0.2. Of these variables ~~the variables found to be significant in~~ bi-variable ~~analysi~~s: maternal age, maternal educational status, ANC follow up and knowledge about COVID-19 were statistically significant to adherence with COVID-19 preventive practice in the multiple ~~multivariable~~ logistic regression.

Study participants whose age ≤ 24 years were 2.89 times more likely to have good adherence with COVID-19 preventive practice as compared to participants whose age group of ≥35 years (AOR = 2.89, 95% CI: 1.37, 6.10). Respondents who had an educational level of secondary school and college and above were 2.95, and 4.57 times more likely to have good adherence with COVID-19 preventive practice as compared to women's who cannot ~~able to~~ read and write respectively (AOR=2.95, 95% CI: 1.58, 5.53), (AOR= 4.57, 95% CI: 2.42, 8.62). The odds of good adherence with COVID-19 preventive practice were 2.95 times higher among women who had ANC follow-up as compared to their counterparts (AOR=2.95, 95% CI: 1.35, 6.46). Pregnant women who had adequate knowledge about COVID-19 were 1.7 times more likely to adhere to COVID-19 preventive practice than pregnant women who had inadequate knowledge about COVID-19 (AOR= 1.70, 95% CI: 1.20, 2.41) **(Table 5).**

Discussion

This study has attempted to assess adherence with COVID-19 preventive practice and associated factors among pregnant women in Gondar city, northwest Ethiopia, 2021. The study result showed that only 44.8% (95% CI: 41.3, 48.7) of the study participants had good adherence with COVID-19 preventive practice. These results are comparable with studies conducted in Ghana 46.6% (27), Egypt 42.86% (29), Wollega zone, Ethiopia 43.6% (28), and Debre Tabor, Ethiopia 47.6% (24).

This finding is lower than studies conducted in Nigeria 79.2% (30) and studies done in some parts of Ethiopia such as Gurage zone, 76.2% (26), Debre Berhan, 56.1% (31). The possible reason for this discrepancy could be due to the difference in the study setting, variation in measuring outcome variable, and sociodemographic characteristics of the study participants. All the above-mentioned studies were institution-based cross-sectional studies. Women who came for ANC follow-up can get information about COVID-19 and are highly likely to practice it. Studies in Nigeria and Debre Berhan used mean score and 80% as a cut of point to say participants had good adherence respectively. Whereas, in this study, 100 % was used as a cut of point to measure the outcome

variable. In addition, 91% and 87.2% of study participants in Nigeria attained at least primary education and had good knowledge of COVID-19 infections respectively. However, in this study, more than one-third (34.8%) of pregnant women had no formal education and 63.7% had adequate knowledge about COVID-19.

However, the result of this study is higher than the study in Ebonyi, state, Nigeria 30.3% (32). The possible explanation for the discrepancy might be due to differences in the time gap of the study and study setting. As time goes community awareness creation and Information dissemination strategies also improved and it might have a positive impact on preventive practice. In addition, 39.1% of study participants in Ebonyi, state were rural dwellers while this study was conducted on urban residents. It has been evidenced that pregnant women living in urban areas were highly likely to practice preventive measures (27,28).

In this study, maternal age is one of the variables significantly associated with good adherence to COVID-19 preventive practice. Study participants whose age ≤24 years were 2.89 times more likely to have good adherence with COVID -19 preventive practice as compared to participants whose age was≥35 years. This finding was supported by a study conducted in Nigeria (30) and studies in different parts of Ethiopia, Wollega zones, (28), Gurage zone, (26). This might be due to younger women having access to information on COVID-19 (risk, mode of transmission, and way of preventive measures) via social media and mass media. Also, it might be younger women have better educational attainment than older women. Pregnant women who had a higher level of education have good adherence with COVID -19 preventive practice. On the contrary, studies conducted in Ghana (27) and Debre Berhan, Ethiopia (31), as age increase the odd of good preventive practice also increase.

The study also revealed that women with educational level of secondary school and, college and above were 2.95 and 4.57 times more likely to have good adherence with COVID-19 preventive practice as compared to women's who cannot able to read and write respectively. This finding was supported by studies conducted in Ghana (27), Debre Tabor, Ethiopia (24). This might be due to the fact that educated people can access information about COVID -9 including its prevention method from different sources than those unable to read and write. In addition, educated people might have a better understanding of preventive measures and consequences associated with not doing this preventive practice.

ANC follow up also one of the important predictors of good adherence to COVID-19 preventive practice. The odds of good adherence with COVID-19 preventive practice were 2.95 times higher among women who had ANC follow-up as compared to their counterparts. This might be pregnant women who had ANC follow-up have a chance to get information about the pandemic from the health care providers during their visit. Study showed that obtaining COVID-19 education at a health facility was significantly associated with adequate knowledge on COVID-19 prevention (27).

This study also showed that there was a significant association between the knowledge of the respondents and adherence to COVID-19 preventive practice. Pregnant women who had adequate knowledge about COVID-19 were 1.7 times more likely to adhere to COVID-19 preventive practice than pregnant women who had inadequate knowledge about COVID-19. This finding was supported by studies conducted in Debre Berhan (31) and Debre Tabor, Ethiopia (24). This might be due to women who had prior knowledge about the pandemic including the benefits of preventive measures may apply it to protect themselves and their families against the pandemic.

Limitation of the study

The cross-sectional nature of the study design might not possible to infer the cause and effect relationship between pregnant women's adherence with COVID-19 preventive practice and its associated factors. There might be also a social desirability bias.

Conclusion

In this study, adherence with COVID-19 preventive practice among pregnant women is low. Maternal age, maternal occupational status, ANC follow up and knowledge about COVID-19 were factors affecting adherence with COVID-19 preventive practice. Therefore, it is important to strengthen the health education of pregnant women about COVID-19 preventive practice through different media. In addition, empowering women to attain ANC and special consideration should be given to women who had no formal education.

Declarations

List of abbreviations

ANC: Antenatal Care; AOR: Adjusted Odds Ratio; CI: Confidence Interval; COR: Crude Odds Ratio; COVID-19: Corona virus disease 2019; SARS-CoV-2: Severe Acute Respiratory Syndrome Coronavirus 2; WHO: World Health Organization

Consent to publish: Not applicable

Data Availability Statement: All relevant data are within the manuscript and its Supporting information files.

Competing interests: The authors declare that they have no competing interests

Funding: no fund was received for this work

Authors' contributions

Conceptualization: Wubedle Zelalem Temesgan

Data curation: Wubedle Zelalem Temesgan, Mastewal Belayneh Aklil, Henok Solomon Yacob, Esubalew Tsega Mekonnen, Elias Derso Tegegne, Esubalew Binega Tesfa, Eshetie Melkie Melese, Tewodros Seyoum

Formal analysis: Wubedle Zelalem Temesgan, Mastewal Belayneh Aklil, Henok Solomon Yacob, Esubalew Tsega Mekonnen, Elias Derso Tegegne, Esubalew Binega Tesfa, Eshetie Melkie Melese, Tewodros Seyoum

Investigation: Wubedle Zelalem Temesgan

Methodology: Wubedle Zelalem Temesgan, Mastewal Belayneh Aklil, Henok Solomon Yacob, Esubalew Tsega Mekonnen, Elias Derso Tegegne, Esubalew Binega Tesfa, Eshetie Melkie Melese, Tewodros Seyoum

Supervision: Wubedle Zelalem Temesgan, Mastewal Belayneh Aklil, Henok Solomon Yacob, Esubalew Tsega Mekonnen, Elias Derso Tegegne, Esubalew Binega Tesfa, Eshetie Melkie Melese, Tewodros Seyoum

Validation: Wubedle Zelalem Temesgan, Mastewal Belayneh Aklil, Henok Solomon Yacob, Esubalew Tsega Mekonnen, Elias Derso Tegegne, Esubalew Binega Tesfa, Eshetie Melkie Melese, Tewodros Seyoum

Visualization: Wubedle Zelalem Temesgan, Mastewal Belayneh Aklil, Henok Solomon Yacob, Esubalew Tsega Mekonnen, Elias Derso Tegegne, Esubalew Binega Tesfa, Eshetie Melkie Melese, Tewodros Seyoum

Writing- original draft: Wubedle Zelalem Temesgan, Mastewal Belayneh Aklil, Henok Solomon Yacob, Esubalew Tsega Mekonnen, Elias Derso Tegegne, Esubalew Binega Tesfa, Eshetie Melkie Melese, Tewodros Seyoum

Writing-review and editing: Wubedle Zelalem Temesgan, Mastewal Belayneh Aklil, Henok Solomon Yacob, Esubalew Tsega Mekonnen, Elias Derso Tegegne, Esubalew Binega Tesfa, Eshetie Melkie Melese, Tewodros Seyoum

Final approval of the version to be published: Wubedle Zelalem Temesgan, Mastewal Belayneh Aklil, Henok Solomon Yacob, Esubalew Tsega Mekonnen, Elias Derso Tegegne, Esubalew Binega Tesfa, Eshetie Melkie Melese, Tewodros Seyoum

Acknowledgments

We would like to thank the university of Gondar for Ethical approval. We would also like to extend our gratitude to each Keeble administrative, data collectors, supervisor and study participants.

Reference

1. Awadasseid A, Wu Y, Tanaka Y, Zhang W. Initial success in the identification and management of the coronavirus disease 2019 (COVID-19) indicates human-to-human transmission in Wuhan, China. Int J Biol Sci , 2020;16(11):1846.

2. Denis M, Vandeweerd V, Verbeeke R, Laudisoit A, Reid T, Hobbs E, et al. Covipendium: Information available to support the development of medical countermeasures and interventions against COVID-19. Transdiscipl Insights. 2021;4(1):1–296.

3. World Health Organization coronavirus disease (COVID-19) Pandemic,2020.

4. Li Q, Guan X, Wu P, Wang X, Zhou L, Tong Y, et al. Early Transmission Dynamics in Wuhan, China, of Novel Coronavirus–Infected Pneumonia. N Engl J Med. 2020 Mar 26;382(13):1199–207.

5. Abdel Wahed WY, Hefzy EM, Ahmed MI, Hamed NS. Assessment of Knowledge, Attitudes, and Perception of Health Care Workers Regarding COVID-19, A Cross-Sectional Study from Egypt. J Community Health. 2020 Dec 1;45(6):1242–51.

6. Ortiz-Prado E, Simbaña-Rivera K, Gómez- Barreno L, Rubio-Neira M, Guaman LP, Kyriakidis NC, et al. Clinical, molecular, and epidemiological characterization of the SARS-CoV-2 virus and the Coronavirus Disease 2019 (COVID-19), a comprehensive literature review. Vol. 98, Diagnostic Microbiology and Infectious Disease. Elsevier Inc.; 2020. p. 115094.

7. Wondimu W, B Girma .Challenges and silver linings of COVID-19 in Ethiopia–short review. journal of multidisciplinary,2020.

8. Shaibu Amiri BED Graduate Josiah A, Akram M. COVID-19: THE CHALLENGES OF THE HUMAN LIFE,2021.

9. Worldometer report on coronavirus disease-19 on November 20/2021. https: //www.worldometers.info/coronavirus/.

10. World Health Organization:Advice for the public,coronavirus disease (COVID-19)

11. Baye K. COVID-19 prevention measures in Ethiopia: current realities and prospects. 2020

12. Rasmussen SA, Smulian JC, Lednicky JA, Wen TS, Jamieson DJ. Coronavirus Disease 2019 (COVID-19) and pregnancy: what obstetricians need to know. Am J Obstet Gynecol. 2020;222(5):415–26.

13. Zhao X, Jiang Y, Zhao Y, Xi H, Liu C, Qu F, et al. Analysis of the susceptibility to COVID-19 in pregnancy and recommendations on potential drug screening. Eur J Clin Microbiol Infect Dis. 2020;39(7):1209–20.

14. Mullins E, Evans D, Viner RM, O'Brien P, Morris E. Coronavirus in pregnancy and delivery: rapid review. Ultrasound Obstet Gynecol. 2020;55(5):586–92.

15. Ellington S, Strid P, Tong VT, Woodworth K, Galang RR, Zambrano LD, et al. Characteristics of women of reproductive age with laboratory-confirmed SARS-CoV-2 infection by pregnancy status-United States, January 22-June 7, 2020. Obstet Gynecol Surv. 2020;75(11):664–6.

16. Musa IR. Risk Factors Contributing in Increased Susceptibility and Severity of COVID-19 Infection during Pregnancy. Open J Obstet Gynecol. 2020;10(09):1197–215.

17. Di Mascio D, Khalil A, Saccone G, Rizzo G, Buca D, Liberati M, et al. Outcome of coronavirus spectrum infections (SARS, MERS, COVID-19) during pregnancy: a systematic review and meta-analysis. Am J Obstet Gynecol MFM. 2020;2(2):100107.

18. Ciarambino T, Crispino P, Giordano M. Viral Infection, COVID-19 in Pregnancy and Lactating Women: What Is Known? Covid. 2021;1(1):230–45.

19. RCM|Royal college of Midwives and the RCOG|Royal college of Obstetricians & Gynecologists. Coronavirus (COVID-19) Infection in Pregnancy:Information for healthcare professionals Version 11,2020:1–68.

20. Society for Maternal-Fetal Medicine (SMFM) Statement: SARS-CoV-2 Vaccination in Pregnancy.

21. Smith DD, Pippen JL, Adesomo AA, Rood KM, Landon MB, Costantine MM. Exclusion of Pregnant Women from Clinical Trials during the Coronavirus Disease 2019 Pandemic: A Review of International Registries. Am J Perinatol. 2020;37(8):792–9.

22. Riley LE, Beigi R, Jamieson DJ, Hughes BL, Swamy G, Eckert LON, et al. Vaccinating Pregnant and Lactating Patients Against COVID-19 Summary of Key Information and Recommendations COVID-19 Infection Risk in Pregnancy COVID-19 Vaccines in Development. Am Coll Obstet Gynecol. 2021;19:1–20.

23. Federal Demographic Republic of Ethiopia Central Statistical Agency Population Projection of Ethiopia for All Regions.

24. Ayele AD, Mihretie GN, Belay HG, Teffera AG, Kassa BG, Amsalu BT. Knowledge and practice to prevent COVID-19 and its associated factors among pregnant women in Debre Tabor Town Northwest Ethiopia, a community-based cross-sectional study. BMC Pregnancy Childbirth. 2021;21(1):1–12.

25. Abeya SG, Barkesa SB, Sadi CG, Gemeda DD, Muleta FY, Tolera AF, et al. Adherence to COVID-19 preventive measures and associated factors in Oromia regional state of Ethiopia. PLoS One , 2021;16(10):

26. Fikadu Y, Yeshaneh A, Melis T, Mesele M, Anmut W, Argaw M. Covid-19 preventive measure practices and knowledge of pregnant women in guraghe zone hospitals. Int J Womens Health. 2021;13:39–50.

27. Kumbeni MT, Apanga PA, Yeboah EO, Lettor IBK. Knowledge and preventive practices towards COVID-19 among pregnant women seeking antenatal services in Northern Ghana. PLoS One. 2021;16(6 June):1–11.

28. Besho M, Tsegaye R, Yilma MT, Kasaye HK, Tolossa T, Hiko N, et al. Knowledge, attitude and practice toward corona virus infection among pregnant women attending antenatal care at public hospitals in three wollega zones, ethiopia. Int J Gen Med. 2021;14:3563–73.

29. Mohamed Moselhy M, Attaia Z. Knowledge, Attitude and Preventive Practices toward Current Pandemic COVID-19 Among Pregnant Women. Egypt J Nurs Heal Sci.

2021;2(2):176–203.

30. Aduloju OP, Okunola TO, Adefisan AS, Aduloju T. Knowledge, attitude and practice of preventive measures against coronavirus disease 2019 among pregnant women in a tertiary health facility in southwest Nigeria. Int J Clin Obstet Gynaecol. 2021;5(3):101–7.

31. Silesh M, Demisse TL, Taye BT, Desta K, Kitaw TM, Mekuria AD, et al. Compliance with COVID-19 Preventive Measures and Associated Factors Among Women Attending Antenatal Care at Public Health Facilities of Debre Berhan Town, Ethiopia. Risk Manag Healthc Policy. 2021;14:4561–9.

32. Nwafor JI, Aniukwu JK, Anozie BO, Ikeotuonye AC, Okedo-Alex IN. Pregnant women's knowledge and practice of preventive measures against COVID-19 in a low-resource African setting. Int J Gynecol Obstet, 2020 Jul 1 150(1):121–3.

List of tables

Table 1: Sociodemographic characteristics of pregnant women in Gondar city, northwest Ethiopia 2021(n=663)

**Variables**	**Frequency**	**Percent (%)**
**Maternal age**		
≤24 years	43	6.5
25-34 years	381	57.5
≥35 years	239	36
**Religion**		
Orthodox	440	66.4
Muslim	178	26.8
Protestant	41	6.2
Catholic	4	0.6
**Maternal educational status**		
Unable to read and write	71	10.7
Able to read and write	160	24.1
Elementary(1-8)	106	16
Secondary School(9-12)	167	25.2
College and above	159	24
**Maternal occupation**		
Housewife	273	41.2
Civil servant	170	25.6
Private Business	186	28.1
Farmer	14	2.1
Others*	20	3
**Marital status**		
Married	623	94
Unmarried	40	6

**Husband educational status (n=623)**		
Unable to read and write	33	5.3
Able to read and write	174	27.9
Elementary(1-8)	51	8.2
Secondary School(9-12)	196	31.5
College and above	169	27.1
**Husband occupation (n=623)**		
Civil servant	209	33.5
Private Business	352	56.5
Farmer	56	9.0
Others**	6	1.0
**Number of family living together**		
≤2	106	16.0
3-4	300	45.2
≥5	257	38.8
**Average monthly income**		
<5000 ETB	177	26.7
5000-10000 ETB	323	48.7
>10000 ETB	163	24.6

Others*; daily laborer and student, Others**; daily laborer and unemployed, ETB; Ethiopian birr

Table 2: Obstetric and reproductive characteristics of pregnant women in Gondar city, northwest Ethiopia, 2021 (n=663)

Variables	Frequency	Percent (%)
**Gravidity**		
Primigravida	134	20.8
Multigravida	529	79.2
**Number of alive children**		
No alive children	141	21.3
<3	345	52
≥3	177	26.7

**ANC follow up**		
Yes	619	93.4
No	44	6.6
**Number of ANC follow up (n=619)**		
≤3	444	71.7
>3	175	28.3
**Condition of pregnancy**		
Planned	601	90.6
Unplanned	62	9.4
**History of previous adverse pregnancy outcome**		
Yes	121	18.3
No	542	81.7
**History of chronic illness**		
Yes	132	19.9
No	531	80.1

Table 3: knowledge of pregnant women about COVID-19 in Gondar city, northwest Ethiopia 2021(n=663)

**Knowledge item**	**Correct response n (%)**	**Incorrect response n (%)**
Ever heard about COVID-19	660 (99.5)	3 (0.5)
COVID-19 is viral disease	561 (84.6)	102 (15.4)
Respiratory droplets and close contact are the main transmission route.	578 (87.2)	85 (12.8)
All people are generally susceptible to COVID-19	459 (69.2)	204 (30.8)
Dry cough is a symptom of COVID-19	521 (78.6)	142 (21.4)
Fever is a symptom of COVID-19	405 (61.1)	258 (38.9)
Headache is a symptom of COVID-19	502 (75.7)	161 (24.3)
Sore thorat is a symptom of COVID-19	347 (52.3)	316 (47.7)

Runny nose is a symptom of COVID-19	355 (53.5)	308 (46.5)
Difficulty of breathing is a symptom of COVID-19	557 (84)	106 (16)
Stay at home and wearing face mask can prevent COVID-19	630 (95)	33 (5)
People with co-existing disease has poor prognostic outcome	427 (64.4)	236 (35.6)
Person with COVID-19 can transmit the virus to others without developing sign	437 (65.9)	226 (34.1)
Pregnant women are at high risk than others	527 (79.5)	136 (20.5)

Table 4: Adherence with COVID-19 preventive practice among pregnant women in Gondar city, northwest Ethiopia, 2021

**Preventive Practice**	**Frequency**	**Percent (%)**
**Wash hands with water and soap or with sanitizer**		
Yes	491	74.1
No	172	25.9
**Avoid touching eyes, nose and mouth with unwashed hand**		
Yes	432	65.2
No	231	34.8
**Cover mouth and nose during coughing and sneezing.**		
Yes	508	23.4
No	155	76.6
**Wear face mask in public**		
Yes	599	90.3
No	64	9.7
**Stay at home or in door**		
Yes	501	75.6
No	162	24.4
**Maintain 1 meter distance from others**		

Yes	388	58.5
No	275	41.5

Simple and multiple

Table 5: ~~Bivariable and multivariable~~ logistic regression analysis of factors affecting adherence with COVID 19 preventive practice among pregnant women in Gondar city, northwest Ethiopia, 2021.

**Variables**	**Adherence**		**COR (95%CI)**	**AOR (95% CI)**
	**Good**	**poor**		
**Maternal age**				
≤24	28	15	2.73 (1.39, 5.38)	**2.89 (1.37, 6.10)***
25-34	172	209	1.20 (0.87, 1.67)	1.05 (0.74, 1.52)
≥35	97	142	1	1
**Maternal educational status**				
Unable to read and write	19	52	1	1
Able to read and write	45	115	1.07 (0.57, 2.01)	0.93 (0.53, 1.89)
Elementary(1-8)	38	68	1.53 (0.79, 2.95)	1.36 (0.68, 2.71)
Secondary School(9-12)	91	76	3.28 (1.78, 6.01)	**2.95 (1.58, 5.53)***
College and above	104	55	5.17 (2.79, 9.61)	**4.57 (2.42, 8.62)****
**Marital status**				
Married	272	351	0.46 (0.24, 0.90)	0.56 (0.27, 1.16)
Unmarried	25	15	1	1
**Number of family living together**				
≤2	56	50	1.82 (1.15, 2.87)	0.58 (0.24, 1.39)
3-4	143	157	1.48 (1.05, 2.07)	0.76 (0.45, 1.30)
≥5	98	159	1	1
**Average monthly income**				
<5000	67	110	1	1
5000-10000	142	181	1.29 (0.88, 1.87)	0.98 (0.65, 1.48)

>10000	88	75	1.93 (1.25, 2.97)	1.48 (0.91, 2.41)
**Gravidity**				
Primigravida	74	60	1.69 (1.15, 2.48)	1.42 (0.23, 8.85)
Multigravida	223	306	1	1
**Number of alive children**				
No alive children	77	64	2.35 (1.49,3.70)	1.47 (0.79, 2.71)
<3	160	185	1.69 (1.16, 2.46)	1.26 (0.80, 1.98)
≥3	60	117	1	1
**ANC follow up**				
Yes	287	332	2.94 (1.43, 6.05)	**2.95 (1.35 , 6.46)***
No	10	34	1	1
**Knowledge about COVID-19**				
Adequate knowledge	208	214	1.66 (1.20, 2.29)	**1.70 (1.20, 2.41)***
Inadequate knowledge	89	152	1	1

COR-crude odds ratio AOR-adjusted odds ratio, CI-confidence interval, 1-reference category, *p≤0.05, ** p <0.01

 Click here to access/download;Figure;list of figure.docx

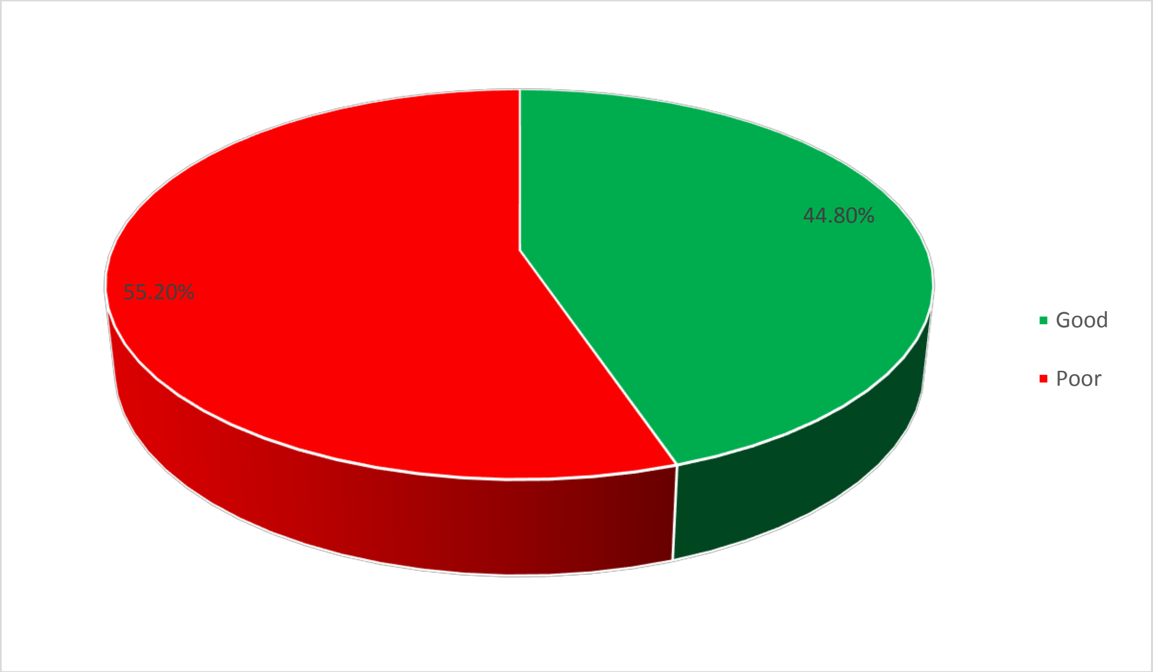

Figure 1: Adherence with COVID-19 preventive practice among pregnant women in Gondar city, northwest Ethiopia, 2021

English version questionnaire

Click here to access/download
Supporting Information
English version questionar.docx

Click here to access/download
Supporting Information
SPSS data.sav
