# Supplementary material for: Adherence to COVID-19 preventive practice and associated factors among pregnant women in Gondar city, northwest Ethiopia, 2021: Community-based cross-sectional study
Source: PLoS One. 2022 Mar 2;17(3):e0264237. doi: 10.1371/journal.pone.0264237 (PMC8890631; doi:10.1371/journal.pone.0264237)
Supplement: Attachment — Submitted filename: Response to Reviewers.docx [file pone.0264237.s004.docx]

**Response to Reviewers	Date: 31/1/2022**

PLOSS ONE

Manuscript number: PONE-D- 21-40668

We would like to say thank you for giving us the opportunity to submit a revised draft of our manuscript titled “**Adherence with COVID-19 preventive practice and associated factors among pregnant women in Gondar city, northwest Ethiopia, 2021: Community-based cross-sectional study”.** We appreciate the time and effort that you have dedicated to provide your valuable feedback on our manuscript. We are grateful to the entire editorial team and peer reviewers for their insightful comments on our paper. We have been able to incorporate changes to reflect most of the suggestions provided by the reviewer.

Here is a point-by-point response to specific comments and concerns.

**Academic Editor’s comments and concerns**

1. Please ensure that your manuscript meets PLOS ONE's style requirements, including those for file naming.

**Response**: Thank you, dear editor, we kindly accepted your request and we did that in the revised manuscript.

2. If you did not receive any funding for this study, please state: “The authors received no specific funding for this work.”

**Response**: Thank you, dear editor, we kindly accepted your request and we have corrected in the revised manuscript as “the authors received no specific funding for this work”. Please see the tack change in the revised manuscript.

3. PLOS requires an ORCID iD for the corresponding author in Editorial Manager on papers submitted after December 6th, 2016. Please ensure that you have an ORCID iD and that it is validated in Editorial Manager.

**Response**: *Thank you for pointing this out, I have already created an ORCID id*

4. PLOS defines a study's minimal data set as the underlying data used to reach the conclusions drawn in the manuscript and any additional data required to replicate the reported study findings in their entirety. All PLOS journals require that the minimal data set be made fully available

***Response****: Thank you very much. We have uploaded the minimal data set as supporting information when we submitted the manuscript. If it is necessary, we have uploaded it again as S1 English version questionnaire and S1 Dataset*.

5. Please review your reference list to ensure that it is complete and correct. If you have cited papers that have been retracted, please include the rationale for doing so in the manuscript text, or remove these references and replace them with relevant current references.

**Response**: Thank you, dear editor, we kindly accepted your request and we have checked all listed references and all are complete, correct and there is no retracted paper.

**Reviewer #1**

1.I would recommend to correctly call a model with only one explanatory variable as simple logistic regression model. A model with more than one explanatory variable is a multiple logistic regression model. It is wrong to call as bivariable (or bivariate) and multivariable or multivariable.

***Response****: Thank you, dear reviewer, for your thoughtful concern. As we tried to review evidence, a multiple or multivariable logistic regression model is a model with more than one explanatory variable with a single binary outcome and we can use interchangeably. However, multivariate implies a statistical analysis with multiple outcomes. Please kindly visit the following articles:*

*1: Distinction between Two Statistical Terms: Multivariable and Multivariate Logistic Regression: doi:10.1093/ntr/ntaa055.*

*2: Multivariable Analysis in Cerebrovascular Research: Practical Notes for the Clinician: DOI: 10.1159/000345491*

2.I proposed few minor corrections in the .pdf appended file.

***Response****: Thank you, as per your kind recommendation, we tried to correct in the revised manuscript. Please kindly see the track changes*.

**Reviewer#2**

MinorComments:
1) A cross-sectional survey of 678 pregnant women in Gondar city can't corroborate the practice practiced by Ethiopia's whole pregnant women population. Mention this in the limitation of the studysection.
***Response****: Thank you, dear reviewer, for your thoughtful concern. We knew that the limitations of the study are those characteristics of design or methodology that impacted or influenced the application or interpretation of the results of the study. Our study finding is generalized to the source population (all pregnant women in Gondar city). But, it is not generalized to Ethiopia's whole pregnant women population. So this not our study limitation*.

2) Introduction: The authors should provide information like mortality rate, incubation, various initial prevention approaches, and diet and natural biomolecules for improving immunity and health (doi: 10.1007/s12088-020-00908-0).

***Response****: The authors would like to thank you for your constructive and helpful feedback. Comment accepted and corrected accordingly. Please kindly see the revised version*.

3) In the introduction, minor information on the variants of COVID-19 and their future challenges can be included i.e. doi: 10.1007/s15010-021-01734-2.

***Response:*** *Thank you for your constructive and helpful feedback. Comment accepted and corrected accordingly. please kindly see the track change.*
3) At least one additional Figure (illustration) may be provided to highlight this study's summary or prospect.

***Response:*** *Thank you for your suggestion. We have agreed with your suggestion and corrected in the revised manuscript. Please kindly see the revised manuscript.*

**Reviewer #3:** 
1. Please provide some information on the effect of diet to boost the immunity of pregnant woman towards the treatment of Covid-19 and their variant in the discussion section and the following paper may be followed and cited; Indian Journal of Microbiology, 2020, volume 60, pages 420–429.

***Response:*** *Thank you for your constructive and helpful feedback. Comment accepted and we incorporate it in the introduction section by considering it is the ideal section . Please kindly see the track change.*

2. I would recommend few more figure to describe your explanations instead of tables.

***Response****: Thank you, dear reviewer, we have amended it accordingly. Please kindly see the revised manuscript.*

*Best regards!*

Wubedle Zelalem Temesgan
